# Molecular Characterization of *Blastocystis* sp. in *Camelus bactrianus* in Northwestern China

**DOI:** 10.3390/ani11113016

**Published:** 2021-10-20

**Authors:** Xin Yang, Yunhui Li, Yuxin Wang, Junwei Wang, Peng Lai, Yuan Li, Junke Song, Meng Qi, Guanghui Zhao

**Affiliations:** 1 College of Veterinary Medicine, Northwest A&F University, Xianyang 712100, China; xinyang@nwafu.edu.cn (X.Y.); yhlivet@163.com (Y.L.); 2019@nwafu.edu.cn (Y.W.); w13186039897@163.com (J.W.); L1593414756@nwafu.edu.cn (P.L.); Liyy0915@nwafu.edu.cn (Y.L.); sjk7998@163.com (J.S.); 2 College of Animal Science, Tarim University, Alar 843300, China

**Keywords:** *Blastocystis* sp., *Camelus bactrianus*, prevalence, subtyping, northwestern China

## Abstract

**Simple Summary:**

Knowledge for the distribution and genetic diversity of *Blastocystis* sp. can provide novel insights for the prevention and control of this parasite. The present study first reported the occurrence of *Blastocystis* infection in *Camelus bactrianus*, an important economic animal in northwestern China. We found the existence of eight *Blastocystis* subtypes in *C. bactrianus*, indicating potential risks and transmission of *Blastocystis* sp. for *C. bactrianus*.

**Abstract:**

*Blastocystis* sp. is an important zoonotic protist in humans and various animals with worldwide distribution. However, there have been no data on the occurrence of *Blastocystis* sp. in *C. bactrianus*, an important economic animal in northwestern China. In the present study, a PCR-sequencing tool based on the SSU rRNA gene was applied to investigate the prevalence and genetic diversity of *Blastocystis* sp. in 638 faecal samples from *C. bactrianus* in 21 sampling sites within three main breeding areas (Gansu, Inner Mongolia and Xinjiang) in northwestern China. The total prevalence of *Blastocystis* sp. was 21.8% (139/638) in *C. bactrianus*, with the infection rates of 29.5% (18/61), 50.0% (14/28) and 19.5% (107/549) for animals aged <2 years, 2–6 years and >6 years, respectively. Significant differences in prevalence were detected among *C. bactrianus* from three geographic areas (χ^2^ = 19.972, *df* = 2, *p* < 0.001) and all sampling sites (χ^2^ = 104.154, *df* = 20, *p* < 0.001). A total of 16 of 21 sampling sites were positive for *Blastocystis* sp., with the prevalence ranging from 7.7% to 70.6%. Sequence analysis of the SSU rRNA gene identified eight subtypes in *C. bactrianus* in the present study, including seven animal adapted subtypes (ST10, ST14, ST21, ST24, ST25, ST26 and ST30) and one potentially novel subtype, with ST10 being the dominant one. To the best of our knowledge, this study provides the first insight for the occurrence and genetic make-up of *Blastocystis* sp. in *C. bactrianus* and contributes to the understanding of the transmission of *Blastocystis* infection in *C. bactrianus* in China.

## 1. Introduction

*Blastocystis* sp. is a common gastrointestinal protist in humans and various animals with worldwide distribution [1,2]. Although there exists controversy on the pathogenicity of *Blastocystis* sp. [3], it is estimated that over 1 billion people are colonized/infected with *Blastocystis* sp. [4]. Meanwhile, co-infections of *Blastocystis* sp. and zoonotic gastrointestinal parasitic pathogens were commonly found in humans. For example, one study found 54 of 1359 schoolchildren infected with at least two of the enteroparasites *Blastocystis* sp., *Cryptosporidium* spp. and *Giardia duodenalis* in Spain [5]. Co-infections of *Blastocystis* sp. and *G. duodenalis*/*Cryptosporidium* spp. were reported in 28 of 255 children in a cross-sectional study in Colombia [6], and mixed infections of *Blastocystis* sp. and *G. duodenalis* were found in 22 of 261 indigenous children in an epidemiological study in Colombian Amazon Basin [7]. *Blastocystis* sp. also has been recognized to be possibly related to Irritable Bowel Syndrome (IBS) in patients, causing a series of gastrointestinal symptoms, e.g., diarrhea, nausea and abdominal pain [7,8,9], and this protist was also likely relevant to the development of colon cancers [10,11]. Recent studies believed that *Blastocystis* sp. would be a common intestinal microbiota highly associated with microbial diversity of intestines and immune state of hosts [12,13].

Understanding the distribution and genetic diversity of pathogens can provide novel insights for the prevention and control of parasitic infections and diseases. Currently, *Blastocystis* sp. was classified into 32 subtypes (STs) based on intra-variations within sequences of the SSU rRNA gene [14,15,16,17,18]. By reviewing subtypes ST1–ST26, it is recognized that ST18–ST20 and ST22 are not recommended in future analysis due to the possible existence of molecular chimaera within these subtypes [15]. Except for ST9, all the remaining subtypes have been reported in animals [19,20]. Significantly, ST1–ST8, ST10, ST12 and ST14 were also found in humans, suggesting a potential zoonotic transmission route between humans and animals of these subtypes [18,21].

*Camelus bactrianus* is a common camel species mainly found in central Asia, western China and India [22,23]. As a ship of the desert, *C. bactrianus* is not only an indispensable transport tool in desert and semi-desert areas, but is also an important economic animal in northwestern China, with a total number of about 405,300 in 2019 [24,25,26]. Under the grazing condition, *C. bactrianus* is easily to be infected with parasitic pathogens, and several zoonotic pathogens (e.g., *Echinococcus granulosus*, *Cryptosporidium* spp., *Toxoplasma gondii*, *G. duodenalis* and *Enterocytozoon bieneusi*) identified in this animal also have important implications for human health [27,28,29,30,31,32].

Though there have been no reports on the occurrence of *Blastocystis* sp. in *C. bactrianus*, *Blastocystis* infection has been detected in *C. dromedarius*, with the prevalence of 24.0–25.0% [33,34,35], and six subtypes, including three zoonotic subtypes (ST1, ST3 and ST5) and three animal adapted subtypes (ST10, ST14 and ST15) [33,34]. To understand the infection status of *Blastocystis* sp. in *C. bactrianus*, the present study explored the colonization frequency and subtype distribution of *Blastocystis* sp. in *C. bactrianus* from three main breeding areas in northwestern China, and assessed the zoonotic potential of this protist in *C. bactrianus*.

## 2. Materials and Methods

### 2.1. Sampling

**Figure 1:**
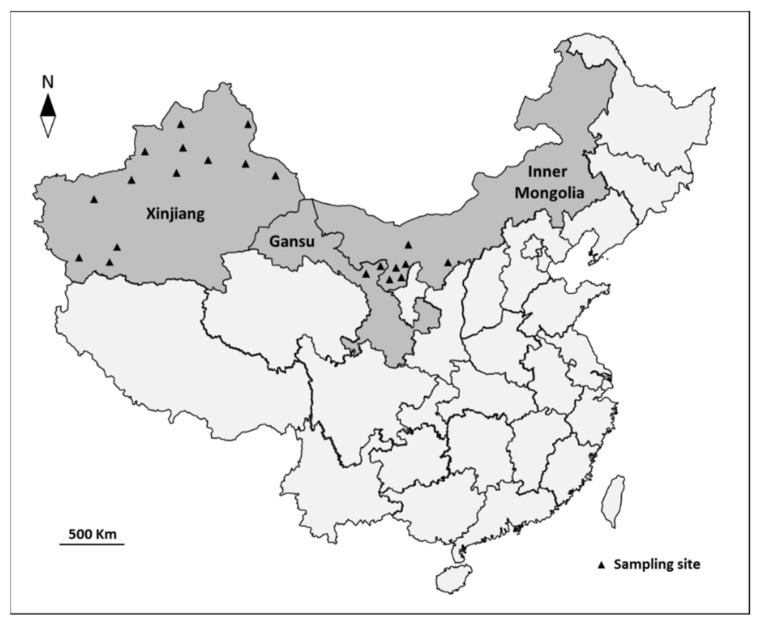
Geographical distribution of sampling sites in the present study. Each black triangle represents one sampling site.

From July 2016 to October 2019, a total of 638 faecal samples of *C. bactrianus* were collected from 21 sampling sites in Gansu, Inner Mongolia and Xinjiang, northwestern China (Figure 1), including 61, 28 and 549 samples from animals aged < 2 years, 2–6 years and >6 years, respectively. All investigated animals were healthy in the present study, and the faecal consistency was normal without obvious diarrhea or other symptoms. All samples were placed into separate bags marked with basic information (e.g., geographic area, sampling site, age), immediately transported to the parasitology laboratory of Northwest A&F University under cool condition, and then kept in 2.5% potassium dichromate at 4 °C for further study.

### 2.2. Genomic DNA Extraction

Each faecal sample was washed three times with distilled water to remove potassium dichromate, and then used for genomic DNA (gDNA) extraction. The E.Z.N.A. Stool DNA kit (Omega, Norcross, GA, USA) was used to isolate gDNA sample from approximately 300 mg faeces of each sample according to the manufacturer, and extracted gDNA samples were kept at −20 °C.

### 2.3. PCR Amplification and Cloning

The occurrence of *Blastocystis* sp. was detected using a PCR targeting an ~500 bp fragment of the SSU rRNA gene using primers Blast505F (5′-GGAGGTAGTGACAATAAATC-3′) and Blast998R (5′-TGCTTTCGCACTTGTTCATC-3′) previously reported [36]. PCR was conducted in a 25 μL reaction mixture containing 1× *Ex Taq* Buffer (Mg^2+^ free), 2 mM MgCl_2_, 0.2 mM dNTP Mixture, 0.625 U TaKaRa *Ex Taq*, 0.4 μΜ each primer and 1 μL gDNA under the following condition: an initial denaturing at 95 °C for 4 min, followed by 35 cycles (denaturing at 95 °C for 30 s, annealing at 54 °C for 30 s, and extension at 72 °C for 30 s), and a final extension at 72 °C for 5 min. Positive PCR products showed an expected band with the size of ~500 bp visualized under a UV transilluminator after 1% agarose gel electrophoresis. All positive amplicons were purified by using TIANgel Midi Purification Kit, cloned into pMD 19-T vector and transformed into *Escherichia coli* JM109. Then, the positive transformants were confirmed by PCR using the forward primer Blast505F and a universal reverse primer M13-48 of the pMD 19-T plasmid under the above-mentioned reaction condition.

### 2.4. Sequencing and Sequence Analysis

Three positive transformants of each faecal sample were sequenced using the primer Blast505F in PCR amplification by Sangon Biotech (Shanghai, China) using an ABI 730 Autosequencer. The obtained nucleotide sequences were confirmed to be *Blastocystis* SSU rRNA gene by BLAST alignment within NCBI (https://blast.ncbi.nlm.nih.gov/Blast.cgi, accessed on 21 June 2021), and then aligned with reference sequences download from GenBank^TM^ within NCBI by using Clustal X 2.1 (www.clustal.org/, accessed on 21 June 2021). To identify subtypes of *Blastocystis* sp. in *C. bactrianus*, a neighbor-joining (NJ) tree was constructed by using the software MEGA 6.06 [37]. The Kimura 2-parameter model was used in the calculation of substitution rates with the bootstrap evaluation of 1000 replicates [38].

### 2.5. Statistical Analysis

Differences in the prevalence of *Blastocystis* sp. among geographical areas and sampling sites were analyzed by using the χ^2^ test within the software SPSS V18.0 (IBM, New York, NY, USA), and the differences were considered significant when *p* value < 0.05.

### 2.6. Nucleotide Sequence Accession Numbers

Representative nucleotide sequences of the present study have been submitted to GenBank^TM^ under the accession numbers of MZ356395-MZ356411, MZ356413-MZ356415, MZ356418-MZ356456, MZ356458-MZ356472 and MZ356474-MZ356480.

## 3. Results and Discussion

*Blastocystis* sp. is an important zoonotic protist with worldwide distribution. Although many studies have reported the prevalence and genetic variations of *Blastocystis* sp. in humans and various animals [1,2,19,39,40], there is still lack of information on the occurrence and genetic diversity of *Blastocystis* sp. in *C. bactrianus*. In the present study, we are the first to investigate the prevalence and genetic diversity of *Blastocystis* sp. in *C. bactrianus* by using the PCR-sequencing tool targeting the SSU rRNA gene. The total prevalence of *Blastocystis* sp. in *C. bactrianus* in our study was 21.8% (139/638), which was similar to that in *C. dromedaries* in Libya (24.0%) [33] and Egypt (25.0%) [34], sheep in the United Kingdom (23.5%) [33], cattle in Brazil (21.4%) [41] and Indonesia (21.2%) [42], and pigs in China (21.7%) [43], but was lower than that in camels in China (50.0%) [39], goats in China (58.1%) [44], Malaysia (30.9%) [45] and Thailand (94.7%) [46], pigs in Spain (46.6%) [47], China (50.0%) [48] and Australia (100%) [49], and cattle in Japan (54.1%) [50], Lebanon (63.4%) [51] and Indonesia (100%) [52], and higher than that in sheep in China (6.0%) [53] and India (14.0%) [54], goats in Nepal (0.8%) [55], pigs in Australia (15.7%) [56] and Spain (7.5%) [57], and cattle in Iran (9.7%) [58], South Korea (6.7%) [59] and Turkey (11.3%) [60]. The differences in the prevalence are possibly caused by sampling size, detection methods and susceptibilities of livestock to *Blastocystis* sp. [15,61]. Further, *Blastocystis* infection was detected in all three investigated geographic areas in our study, with the highest prevalence in Gansu (38.2%, 39/102), followed by Xinjiang (19.8%, 68/344) and Inner Mongolia (16.7%, 32/192), and significant differences in prevalence were found among these geographic areas (χ^2^ = 19.972, *df* = 2, *p* < 0.001). The differences among regions in our study may be associated with different age groups and sample size collected at each region, as well as geographical and animal management practices among regions. Significant differences in prevalence were also recognized among 21 sampling sites (χ^2^ = 104.154, *df* = 20, *p* < 0.001), and *Blastocystis* sp. was identified in 16 sampling sites, with the highest prevalence in Hotan (70.6%, 12/17) and lowest in Ordosr (7.7%, 1/13) (Table 1).

Meanwhile, *Blastocystis* sp. was found in *C. bactrianus* of all three investigated age groups, with the infection rates of 29.5% (18/61), 50.0% (14/28) and 19.5% (107/549) for animals aged <2 years, 2–6 years and >6 years, respectively (Table 2), indicating lower infection rate in older animals (>6 years) compared with that in younger age animals (<2 years and 2–6 years). However, contrary results were recognized in the colonization of *Blastocystis* sp. in cattle because it was mentioned that age was likely a factor influencing prevalence with younger animals having lower prevalence than older animals [62]. These differences indicated potential divergent distribution and transmission of *Blastocystis* sp. among hosts [3,40].

**Figure 2:**
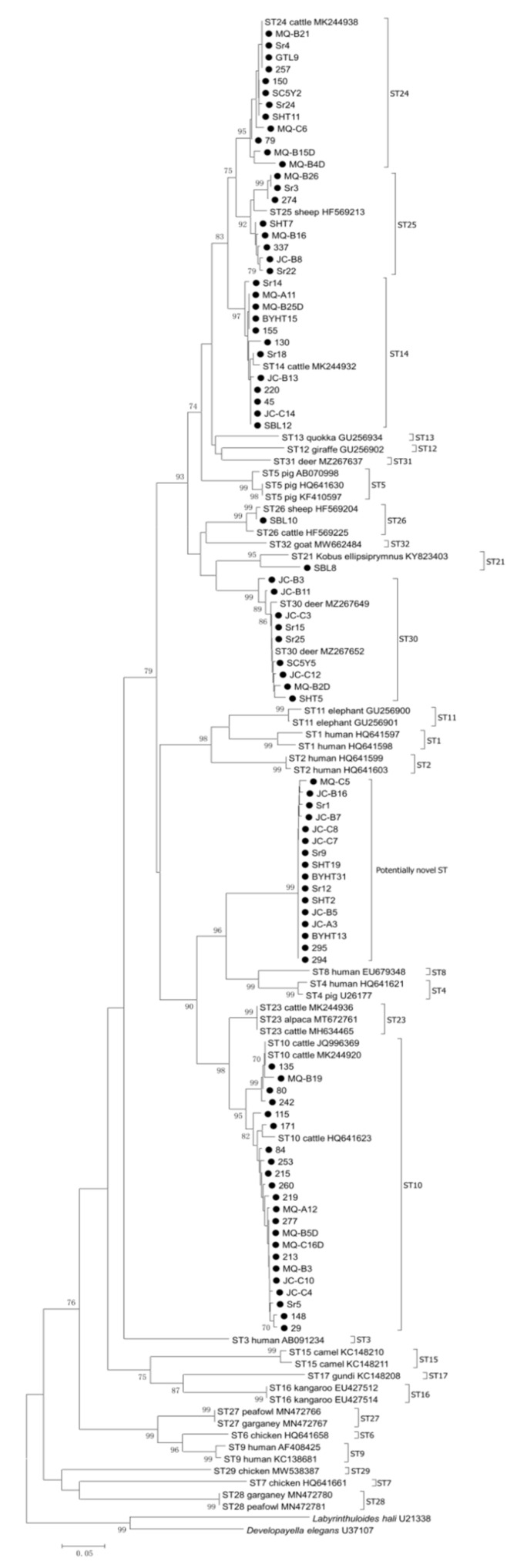
Phylogenetic relationships of *Blastocystis* subtypes in the present study (black filled circles before the sample name) with reference sequences from GenBank^TM^ based on the sequence analysis of the SSU rRNA gene by neighbor-joining analysis using the Kimura 2-parameter model. Bootstrap values (>70) are indicated at the nodes. Scale bar indicates 0.05 nucleotide substitutions/site. *Labyrinthuloides hali* (U21338) and *Developayella elegans* (U37107) are used as the outgroups.

In the present study, all 139 positive samples were sent for sequencing, but we got 134 sequences because five samples failed for sequencing. The length of the 134 obtained sequences ranged from 389 to 409 bp, and a total of 75 haplotypes were identified. Phylogenetic analysis showed eight *Blastocystis* subtypes in *C. bactrianus* in the present study (Figure 2), including seven known subtypes, namely ST10 (49), ST14 (21), ST30 (19), ST24 (14), ST25 (8), ST21 (1) and ST26 (1), and one potentially novel ST (21) (Table 1 and Table 2). Of these, ST10 (36.6%) was the dominant subtype widely distributed in all age groups and most sampling sites (Table 1 and Table 2). Previous studies have also reported the ST10 in various ruminants with worldwide distribution, e.g., cattle, sheep, goats, deer, and camels [19,33,39,44], and one recent study found the existence of ST10 in children in Senegal [21], suggesting the potentially zoonotic transmission of this subtype, which needs further verification in more studies. Besides, ST14 was the second highest frequency (15.7%) in our study, which has also been commonly found in ruminants with worldwide distribution, such as dairy cattle, yaks, water buffalo, takins, alpacas, sheep, goats, antelope, and deer [33,39,61], and also reported in a recent report in children in Senegal [21]. ST24 was a less common subtype with the prevalence of 10.4% in this study, and it has also been found in cattle and deer in the United States [16,38], and in birds in Brazil [14]. Another less common subtype ST25 is identified in 6.0% of samples, which has also been identified in llama in Colombia [17], and in cattle in Spain [63], and chickens in Brazil [18]. In our study, both ST26 and ST21 were found as the lowest frequency (0.8%). ST26, previously identified in dairy cattle [38] and deer [16] in the United States, and in goat, sheep, and cattle in Colombia [17], was found in one camel under two years in Inner Mongolia. ST21, which has been reported in dairy cattle and white-tailed deer in the United States [16,38], and in goats, llama and sheep in Colombia [17], and waterbuck in China [39], was also identified in one camel under two years in Inner Mongolia. Notably, the potentially novel subtype identified in our study was commonly identified in all geographic areas and age groups (Table 1 and Table 2). Sequence alignment indicated that a total of 14 haplotypes were recognized within the SSU rRNA sequences of this potential novel *Blastocystis* subtype in this study, with 37 mutation/indel sites recognized (Supplementary Materials: Table S1). Although zoonotic subtypes were absent in the present study, the frequent distribution of *Blastocystis* subtypes ST10 and ST14 identified in *C. bactrianus* poses a potential threat to the transmission of *Blastocystis* sp. among ruminants in northwestern China.

## 4. Conclusions

The present study first reported *Blastocystis* infection in *C. bactrianus*, with the prevalence of 21.8%, and significant differences in prevalence were observed for animals from different geographic areas and sampling sites. Eight subtypes, including one potentially novel subtype, were identified in *C. bactrianus*. These findings provided fundamental data for the understanding of the transmission of *Blastocystis* sp. in *C. bactrianus* as well as other hosts.

## Figures and Tables

**Table 1:** Occurrence of *Blastocystis* sp. in *Camelus bactrianus* in China.

Geographical Areas	Sampling Sites	No. Examined	No. Positive (%)	Subtypes (No.)
Gansu	Minqin	63	16 (30.2)	ST10 (5); ST14 (3); ST24 (4); ST 25 (2); ST30 (1); PT-ST (1)
	Yongchang	39	20 (51.3)	ST10 (2); ST14 (2); ST 25 (1); ST30 (6); PT-ST (9)
	Subtotal	102	39 (38.2)	ST10 (7); ST14 (5); ST24 (4); ST 25 (3); ST30 (7); PT-ST (10)
Inner Mongolia	Bayanhaote	82	11 (13.4)	ST10 (2); ST14 (2); ST21 (1); ST24 (1); ST26 (1); ST30 (2); PT-ST (2)
	Suhaitu	27	6 (22.2)	ST24 (1); ST25 (1); ST30 (1); PT-ST (3)
	Gilantai	24	2 (8.3)	ST24 (1); ST30 (1)
	Shariburidu	27	12 (44.4)	ST10 (1); ST14 (2); ST24 (2); ST25 (2); ST30 (2); PT-ST (3)
	Tenggeli	19	0 (0)	0
	Ordos	13	1 (7.7)	ST25 (1)
	Subtotal	192	32 (16.7)	ST10 (3); ST14 (4); ST21 (1); ST24 (5); ST25 (4); ST26 (1); ST30 (6); PT-ST (8)
Xinjiang	Bachu	16	0 (0)	0
	Wensu	24	0 (0)	0
	Qitai	19	5 (26.3)	ST10 (1); ST14 (3); ST30 (1)
	Pishan	17	5 (29.4)	ST10 (3); ST14 (2)
	Barkol Kazakh	58	7 (12.1)	ST10 (5); ST14 (1); ST24 (1)
	Qinghe	57	9 (15.8)	ST10 (4); ST24 (2); ST30 (1); PT-ST (2)
	Shihezi	60	17 (28.3)	ST10 (9); ST14 (5); ST24 (1)
	Qapqal Xibe	12	1 (8.3)	ST10 (1)
	Hejing	16	0 (0)	0
	Wushi	10	6 (60.0)	ST10 (5); ST14 (1)
	Tarbagatay	16	0 (0)	0
	Hotan	17	12 (70.6)	ST10 (8); ST30 (4)
	Qira	22	6 (27.3)	ST10 (3); ST24 (1); ST25 (1); PT-ST (1)
	Subtotal	344	68 (19.8)	ST10 (39); ST14 (12); ST24 (5); ST25 (1); ST30 (6); PT-ST (3)
Total		638	139 (21.8)	ST10 (49); ST14 (21); ST21 (1); ST24 (14); ST25 (8); ST26 (1); ST30 (19); PT-ST (21)

Note: PT-ST: potentially novel subtype.

**Table 2:** Age distribution of *Blastocystis* sp. in *Camelus bactrianus* in China.

Age (Years)	No. Examined	No. Positive (%)	Subtypes (No.)
<2	61	18 (29.5)	ST10 (5); ST14 (5); ST21 (1); ST26 (1); ST30 (3); PT-ST (1)
2–6	28	14 (50.0)	ST10 (2); ST14 (1); ST24 (2); ST25 (2); ST30 (1); PT-ST (5)
>6	549	107 (19.5)	ST10 (42); ST14 (15); ST24 (12); ST25 (6); ST30 (15); PT-ST (15)
Total	638	139 (21.8)	ST10 (49); ST14 (21); ST21 (1); ST24 (14); ST25 (8); ST26 (1); ST30 (19); PT-ST (21)

Note: PT-ST: potentially novel subtype.

## Data Availability

Data is contained within the article.

## Supplementary Materials

The following are available online at https://www.mdpi.com/article/10.3390/ani11113016/s1, Table S1: Variations in the SSU rRNA nucleotide sequences among the potentially novel subtype of *Blastocystis* sp. from *Camelus bactrianus* in the present study.
