# Supplementary material for: Molecular Characterization of Blastocystis sp. in Camelus bactrianus in Northwestern China
Source: Animals (Basel). 2021 Oct 20;11(11):3016. doi: 10.3390/ani11113016 (PMC8614482; doi:10.3390/ani11113016)
Supplement: Supplementary file 1 [file animals-11-03016-s001.zip › animals-1395071-supplementary.pdf]

Communication

Molecular Characterization of *Blastocystis* sp. in *Camelus bactrianus* in Northwestern China

Xin Yang [1,†], Yunhui Li [1,†], Yuxin Wang [1,†], Junwei Wang [1], Peng Lai [1], Yuan Li [1], Junke Song [1], Meng Qi [2,*] and Guanghui Zhao [1,*]

[1] College of Veterinary Medicine, Northwest A&F University, Yangling 712100, China; xinyang@nwafu.edu.cn (X.Y.); yhlivet@163.com (Y.L.); 2019@nwafu.edu.cn (Y.W.); w13186039897@163.com (J.W.); L1593414756@nwafu.edu.cn (P.L.); Liyy0915@nwafu.edu.cn (Y.L.); sjk7998@163.com (J.S.)

[2] College of Animal Science, Tarim University, Alar 843300, China

* Correspondence: qimengdz@163.com (M.Q.); zgh083@nwsuaf.edu.cn (G.Z.)

† These authors contributed to the research equally.

Table S1. Variations in the SSU rRNA nucleotide sequences among the potentially novel subtype of *Blastocystis* sp. from *Camelus bactrianus* in the present study.

Nucleotide positions and substitutions	294	JC-B16	JC-B8	BYHT31	JC-B7	JC-C7	BYHT13	MQ-C5	295	SHT19	Sr9	SHT2	Sr1	MQ-B11D
22	-	-	-	-	-	-	-	-	C	-	-	-	-	-
29	T	T	T	T	T	T	T	C	T	T	T	T	T	T
45	C	C	C	C	C	C	C	C	C	C	C	C	C	G
95	C	C	C	C	C	C	C	C	C	C	C	C	C	T
130	C	C	C	C	C	C	C	C	C	C	C	C	T	T
151	A	A	A	A	A	G	A	A	A	A	A	A	A	A
155	A	G	A	A	A	A	A	A	A	A	A	A	A	A
165	A	A	A	A	T	A	A	A	A	A	A	A	A	A
170	T	T	T	T	T	T	T	T	T	T	T	T	T	A
186	C	C	C	C	C	C	C	C	C	C	C	C	C	A
195	T	T	T	T	T	T	T	T	T	T	T	T	T	G
239	C	C	C	C	C	C	C	C	C	C	C	C	C	T
281	A	A	A	A	A	A	A	C	A	A	A	A	A	A
311	A	A	A	T	A	A	A	A	A	A	A	A	A	A
322	T	T	T	T	T	T	T	T	T	T	T	T	T	G
342	A	A	A	A	A	A	A	A	A	A	G	A	A	A
344	T	T	T	T	T	T	T	T	T	G	T	T	T	T
354	T	A	A	T	T	T	T	T	T	T	T	T	T	T
358	G	C	C	G	G	G	G	G	G	G	G	G	G	G
359	A	T	T	A	A	A	A	A	A	A	A	A	A	A
360	G	C	C	G	G	G	G	G	G	G	G	G	G	G
363	G	T	T	G	G	G	G	G	G	G	G	G	G	G
367	G	A	A	G	G	G	G	G	G	G	G	G	G	G
378	T	T	T	T	T	T	T	T	T	T	T	T	T	C
380	C	C	C	C	C	C	C	C	C	C	C	C	C	-
381	A	A	A	A	A	A	A	A	A	A	A	A	A	-
383	T	T	T	T	T	T	T	T	T	T	T	T	T	A
390	A	A	A	A	A	A	A	A	A	A	A	A	A	T
392	G	T	T	A	A	A	A	G	G	G	G	G	A	G
393	A	A	A	G	A	A	A	A	A	A	A	A	A	A
395	T	A	A	T	T	T	T	T	T	T	T	T	T	T
396	-	T	-	-	-	-	-	-	-	-	-	-	-	-
397	-	A	A	-	-	-	-	-	-	-	-	-	-	-
398	G	G	G	G	G	G	G	G	G	G	G	G	G	-
399	T	T	T	T	T	T	T	T	T	T	T	G	T	T
400	T	T	C	T	T	T	T	T	T	T	T	T	T	C
403	G	G	G	G	G	G	G	G	G	G	G	G	G	C
GenBank No.	MZ356453	MZ356444	MZ356415	MZ356447	MZ356443	MZ356442	MZ356449	MZ356441	MZ356455	MZ356452	MZ356451	MZ356450	MZ356445	MZ356457
